# Methylation of the Selected *TP53* Introns in Advanced-Stage Ovarian Carcinomas

**DOI:** 10.7150/jca.94945

**Published:** 2024-05-30

**Authors:** Wiktor Szewczuk, Oksana Szewczuk, Krzysztof Czajkowski, Robert Gromadka, Yan-Gao Man, Maciej Wałędziak, Andrzej Semczuk

**Affiliations:** 1 Department of Pathology, Military Institute of Medicine, Warsaw, Poland.; 2 Department of Gynecology and Gynecologic Oncology, Military Institute of Medicine, Warsaw, Poland.; 3 II ND Department of Obstetrics and Gynecology, Warsaw Medical University, Princess Anna Mazowiecka Hospital, Warsaw, Poland.; 4 Laboratory of DNA Sequencing and Oligonucleotide Synthesis, Institute of Biochemistry and Biophysics, Polish Academy of Sciences, Warsaw, Poland.; 5 Department of Pathology, Hackensack Meridian, Health-Hackensack University Medical Center, Hackensack, New Jersey, NJ, USA.; 6 Department of Surgery, Military Institute of Medicine, Warsaw, Poland.; 7 II ND Department of Gynecological Surgery and Gynecological Oncology, Lublin Medical University, Lublin, Poland.

**Keywords:** *TP53*, intron, DNA, methylation, ovarian cancer, metastasis

## Abstract

**Objective:** Advanced-stage ovarian cancer (OC) is among the most fatal female genital tract neoplasms worldwide. Although different genetic mechanisms have been shown to be involved in ovarian carcinogenesis, the role of *TP53* introns methylation is still unresolved. We performed methylation analysis of introns 1, 3, and 4 of the *TP53* to identify patterns in primary stage III OCs, corresponding metastases, and healthy tissues.

**Methods:** The study involved samples of paraffin-embedded tissues obtained from 80 patients with stage III OCs, who underwent surgery at the Department of Gynecology and Gynecologic Oncology of the Military Institute of Medicine in Warsaw, Poland. Altogether, 40 serous-type G2/3 OCs and 40 endometrioid-type G2/3 OCs were included. From the same patient, metastatic and normal tissues were simultaneously analyzed. As a control group, 80 tissue samples were collected from patients after bariatric operations. Human ovarian cancer A2780 cell line was also investigated. Total genomic DNA was isolated from paraffin-embedded tissue blocks and the methylation analysis was performed by bisulfite DNA conversion, DNA amplification with specific primers, cloning, and DNA sequencing.

**Results:** All of the samples of intron 1 of *TP53* were un-methylated in OCs, metastatic tissues, and in healthy tissues from the same patient. Also, no methylation of *TP53* intron 1 was detected in cells from the human A2780 ovarian cancer cell line and in all samples from control group. In all samples, introns 3 and 4 of the *TP53* were methylated in primary tumors, metastatic tissue, and in healthy tissue from the same patient, in human A2780 ovarian cell line, and in DNA samples from healthy patients. None of the clinicopatholocal features was related to the *TP53* introns methylation status.

**Conclusions:** Our data on *TP53* introns methylation sheds new light on the mechanism of p53 activity for a better understanding of cancer biology. The study suggests the existence of an additional regulation rule of *TP53* activity that involves demethylation-methylation mechanisms. Methylation at introns 3 and 4 may also overall help in protecting *TP53* against damage by viral restrictases or viral DNA integration.

## Introduction

Ovarian cancer (OC) remains the most deadly female genital tract neoplasm worldwide 1. In contrast to other gynecological malignancies, 81% of OC patients were diagnosed at advanced (III or IV due to FIGO) clinical stages. The overall 5-year survival rate for OC women affected by all stages is 45%, whereas for patients with stage III or IV it decreases considerably to only 30% 2, 3.

The lack of highly sensitive and specific molecular and biological markers allowing for early OC detection accounts for the fact that nearly 70% of patients are diagnosed at disseminated disease with unfavorable outcome 2. OC is classified based on histopathological analysis into serous (most common), endometrioid, and clear-cell carcinomas, accounting for 70%, 10%, and 10%, respectively 3.

Tumor protein p53 is a transcription regulator that responds to diverse cellular stresses 4. Moreover, it regulates target genes that induce cell-cycle arrest, cell senescence, DNA repair, and metabolism changes 4-6. It also appears to induce apoptosis through various non-transcriptional cytoplasmic processes. In unstressed cells, p53 is kept in-active essentially through the action of the ubiquitin ligase MDM2 which promotes its degradation. In general, the loss of p53 activity, corresponding with *TP53* alterations, was reported in various human cancers and cell-lines, including primary and metastatic OCs 7-10.

An analysis using limited data sets has suggested that 18-20% of mammalian protein-coding genes use alternative promoters. A study based on a broader data set stated that even 58% of protein-coding transcriptional units had two or even more alternative promoters. These promoters are not only active in different tissues and developmental stages, but they are also associated with various human malignancies, including cancer. The use of these promoters is an important source for regulating gene expression profiling and generating protein diversity 11.

About 81% of all spliced human protein-coding genes have transcriptionally active introns 12-13. Introns constitute up to 95% of the primary gene transcripts of encoding proteins in mammals, though introns do not encode proteins but instead various important genetic functions. Interestingly, introns in the transcribed regions may enhance the expression of its container gene 13. It is worth to emphasize that aberrant expression of intronic noncoding RNAs has been correlated with cancer development and progression 14.

According to Bourdon *et al.* 15, *TP53* has two transcriptional start sites in exon 1, and also contains a transcription initiation site at intron 4. Also, *TP53* has a complex transcriptional expression pattern encoding different p53 mRNA variants *via* alternative splicing and an internal promoter localized at intron 4 15. Reisman *et al.* 16 identified two promoters at the *TP53*, the first one located 100 to 250 bp upstream of the noncoding first exon, whereas the second one (a stronger promoter) is located within the first intron. Interestingly, a novel transcript encoded within the 10-kb intron 1 of the *TP53* has been found out during differentiation of myeloid leukemia cells 17. Moreover, Ribi *et al.* 18 noted that “intron 1 rearrangements cause p53-driven malignancies by both germline and somatic mechanisms and provide an important mechanism of *TP53* inactivation in Li-Fraumeni syndrome”.

The function of intron 3 of the *TP53* is not fully understood yet. Marcel and *co-investigators* 19 suggested that G4 structure in *TP53* intron 3 regulates the splicing of intron 2, leading to differential expression of transcripts encoding distinct p53 isoforms.

CpG islands are short stretches of CpG rich regions that are frequently associated with the promoter region. Aberrant methylation of CpG islands is one of the mechanisms of inactivating tumor suppressor genes in human cancers, and there is a growing amount of evidence suggesting that altered cytosine methylation may play important roles in the development and progression of various human neoplasms 20-24.

The aim of the present study was to investigate the methylation status in three selected introns of the *TP5*3 in primary and metastatic human OCs as well as in normal tissues collected from the same patient. Moreover, human A2780 ovarian cancer cell-line was also analyzed. Finally, we also investigated, as a control group, the methylation of selected *TP53* introns of 80 healthy subjects who have never been affected by cancer.

## Materials and Methods

### Patients and samples

The study group involved samples of paraffin-embedded tissues obtained from 80 patients with OC (mean patient age 63 years old; range 42-80 years old), who underwent surgery at the Department of Gynecology and Gynecologic Oncology of the Military Institute of Medicine in Warsaw, Poland. Material collected at the operation theatre was immediately fixed in buffered formalin (pH 7.4) for routine histopathological assessment. Hematoxylin and eosin-stained slides were prepared from the paraffin-embedded tissue samples and assessed by two independent pathologists to confirm the original diagnosis of ovarian cancer. Altogether, 40 serous-type and 40 endometrioid-type OCs were included 25 (Table 1). We investigated only stage III tumors based on FIGO classification 26. From the same patient, metastatic and normal tissues were simultaneously analyzed. As a control group, 80 samples were collected from healthy volunteers after bariatric operations. Before DNA extraction, samples were carefully assessed pathologically. None of the healthy patients had neoplastic disease in the past. Finally, the human ovarian cancer A2780 cell line (Merck, Darmstadt, Germany) was also investigated.

All patients were informed about the aim of the study and written informed consent was collected.

### Analysis of *TP53 i*ntrons 1, 3, 4 methylation

The methylation of introns 1, 3, and 4 of the *TP53* was analyzed based on the protocol described below.

Before DNA extraction, samples were enriched *via* macro-dissection to increase the content of cells. Total genomic DNA was isolated from paraffin-embedded tissue blocks using the ExtractMe DNA Tissue isolation Kit (Blirt, Gdansk, POLAND) according to the manufacturer's recommendation. The quantity and quality of DNA were analyzed using a DeNovix DS-11 (Wilmington, USA) spectrophotometer. The A260/280 value of genomic DNA showed the purity of the isolated DNA, with values of 1.8 to 2.0 indicating high purity.

Bisulfite DNA conversion was performed using DNA from each sample and a MethylCode Bisulfite Conversion Kit (Invitrogen, Carlsbad, USA) according to the manufacturer's instructions.

Two pairs of gene-specific primers sequences for introns 1, 3, and 4 were designed according to the predicted sequence of the *TP53* gene in NCBI (NC_000017.10) using MethPrimer-Design (www.urogene.org) software. The primer sequences and the product sizes are shown in Table 2.

Amplification of *TP53* introns 1, 3, and 4 was conducted by applying the MyTaqHS Red Mix (Blirt, Gdansk, Poland) with appropriate primers. For intron 1, the thermal cycle profile comprised initial denaturation at 95 °C 1 min, followed by denaturation at 95 ˚C for 15 s, annealing at 62 °C for 15 s, extension at 72 °C for 15 s, and terminal extension at 72 °C for 4 min. For intron 3, the thermal cycle profile comprised initial denaturation at 95 °C for 1 min, followed by denaturation at 95 °C for 15 s, annealing at 58 °C for 15 s, extension at 72 °C for 15 s, and terminal extension at 72 °C for 4 min. For intron 4, the thermal cycle profile comprised initial denaturation at 95 °C for 1 min, followed by denaturation at 95 °C for 15 s, annealing at 56°C for 15 s, extension at 72°C for 15 s, and terminal extension at 72 °C for 4 min. The products were stored at 4°C.

The PCR products were detected after electrophoresis on 1.5% agarose gel using Midori Green nuclear staining dyes (Nippon Genetics Europe GmbH, Duren, Germany). For cloning, products were purified using the ExtractMe DNA Gel-out Kit (Blirt, Gdańsk, Poland).

The ligation between the plasmid vector and PCR product was carefully carried out in a sterile environment using the Qiagen PCR Cloning KIT (Quiagen, Hombrechtikon, Germany). Finally, the vectors were transformed into *E. coli* competent cells with high efficiency (New England BioLabs Ltd, Hitchun, UK). Cells were incubated at 37 °C overnight on LB Agar Miller (A&A Biotechnology, Gdansk, Poland) with ampicillin, IPTG, and X-gal (Blirt, Gdansk, Poland). A blue-white screening colony selection method was used to select a recombinant, white-colored clone, followed by PCR amplification of the colony to confirm cloning with the appropriate gene segments. White colonies with the recombinant plasmid were isolated from the bacteria growing in LB media and cultured in a LB media at 37°C overnight. The plasmid was isolated using a Plasmid Mini DNA Isolation Kit (A&A Biotechnology, Gdańsk, Poland). The result of the recombinant DNA of three introns was examined using agarose gel electrophoresis. Clones containing inserts of the right size were directly sequenced using genetic analyzer AB3130 with T7/SP6 primers and an ABI Prism Big Dye Terminator v3.1 Cycle Sequencing Kit (Abcam, Cambridge, UK). Sequences were analyzed by two investigators (WS, RG).

## Results

Altogether, 80 stage III primary OCs with corresponding metastases as well as healthy tissues from the same patient, were investigated. Moreover, 80 samples of patients, who had never had cancer during their live spans, were analyzed. Finally, the human A2780 ovarian cancer cell-line was also examined.

### TP53 intron 1 methylation

**Figure 1:**
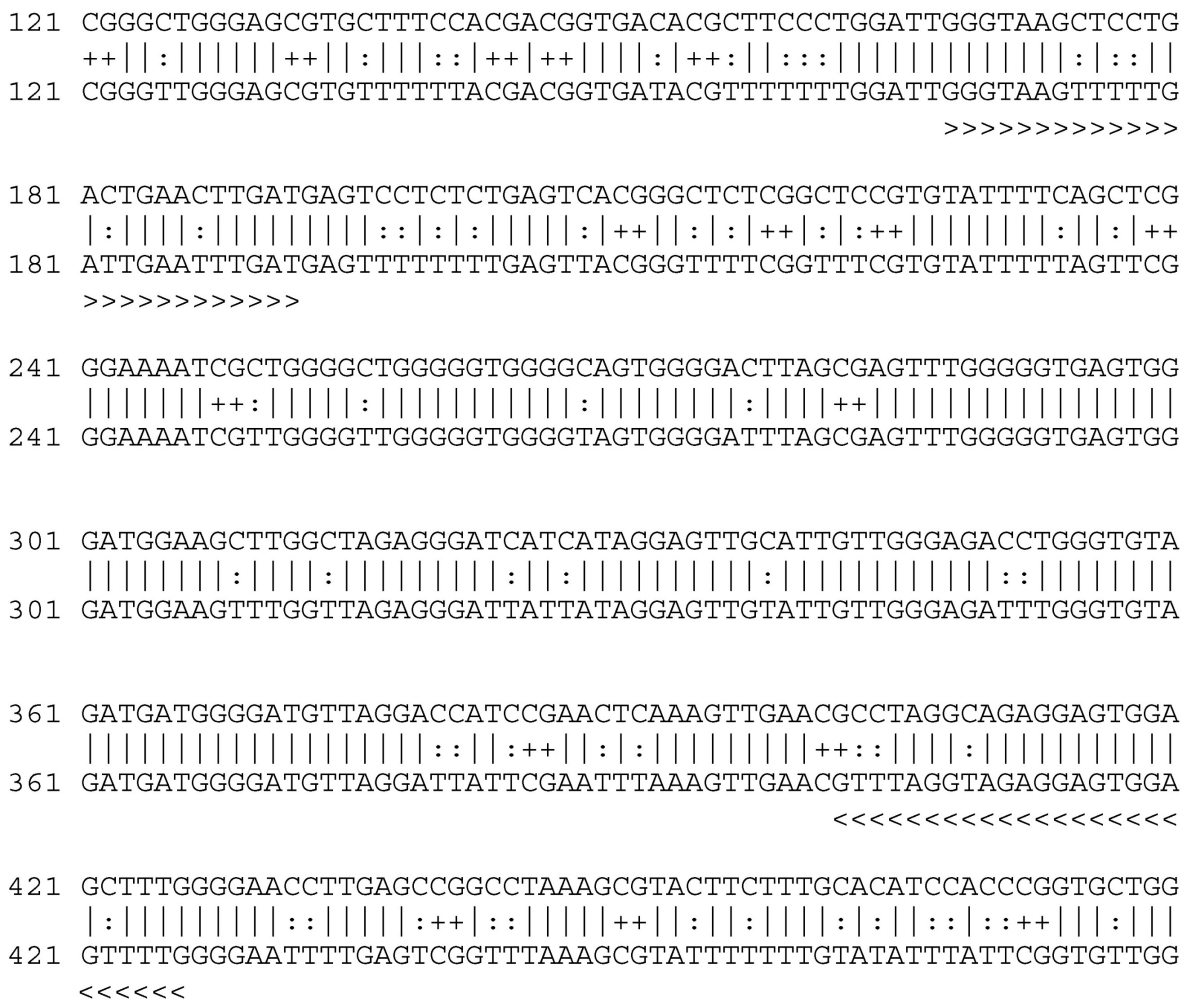
*TP53* intron 1 sequence showing eight CpG pairs investigated (marked yellow). >>>> - primer forward; <<<< - primer reverse.

**Figure 2:**
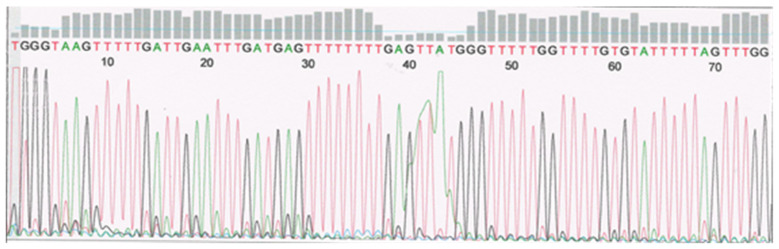
Sample of primary serosum ovarian cancer after sequencing. Intron 1. Unmethylated cytosine replaced with thymine in position 44, 52, 58, 73.

Altogether, eight out of 8 (100%) CpG pairs localized within the intron 1 of the *TP53* were investigated (Fig. 1; Tab. 2). We reported no methylation of the *TP53* intron 1 in primary tumors, metastases, and healthy tissues from the patients. The human A2780 ovarian cancer cell line and 80 healthy patients were all un-methylated at intron 1 of the *TP53* (Fig. 2).

### TP53 intron 3 methylation

We searched for the methylation status within two out of 3 (67%) of the CpG pairs localized within the intron 3 of the *TP53*. Methylation of intron 3 was reported in all primary neoplasms, corresponding metastases, and healthy tissues from patients with OC, in human A2780 ovarian cancer cells, and in 80 tissues obtained from healthy people.

### TP53 intron 4 methylation

PCR-primers were designed to analyze simultaneously intron 4 and exon 4 *TP53* methylation status. Interestingly, one CpG pair was only localized within intron 4 of the *TP53*, whereas 10 pairs were spanned at exon 4 (data not shown).

Intron 4 *TP53* was methylated similarly to intron 3, in all primary neoplasms, corresponding metastases, and healthy tissues from women with OCs, in human A2780 ovarian cancer cells, and in 80 tissues collected from healthy people.

None of the clinicopathological features investigated was related to the *TP53* introns methylation status (data not shown).

## Discussion

Based on the literature review, we found no data reporting the *TP53* intron methylation patterns in advanced-stage human OCs up to now /Pubmed^®^ database/. Interestingly, although methylation of the introns 3 and 4 was present in all samples investigated as well as in human A2780 ovarian cell-line, none of intron 1's eight CpG pairs was methylated. Although FIGO stage III neoplasms were only investigated (normal tissues, primary tumors and corresponding metastases), the study group was large enough to draw final conclusions.

There are several data reporting the *TP53* promoter methylation status in different human neoplasms, including primary human OCs 27-29. Interestingly, Chmelarova and *co-investigators* 27 investigated the *TP53* promoter methylation in OCs compared with normal ovarian tissues. As high as 51.1% of neoplastic samples showed *TP53* promoter methylation, suggesting that it “..may have implications for future chemotherapy based on epigenetic changes.”. Interestingly, there was no *TP53* promoter methylation in 54 endometrioid endometrial carcinomas by the same group of scientists 28. In north Indian population of cervical cancer patients, *TP53* promoter methylation was observed to be significant for oral contraceptive users and for women having age at first sexual intercourse <20 years 29.

Tornaletti and Pfeifer 30 used genomic sequencing to define the complete DNA methylation pattern along exons 5-8 of the human *TP53* suppressor gene. Using nine types of normal human tissue and cell-lines, including skin fibroblasts, keratinocytes, lung epithelial cells, mammary epithelial cells and colonic mucosa cells, they found that *TP53* sequences along exons 5-8 are completely methylated at every CpG pairs, including 46 different sites on both DNA strands. Moreover, *TP53* methylation pattern was tissue-independent 28. They finally assumed that “..given the lack of tissue specificity of methylation, alternative explanations (e.g. targeting of methylated CpG sites by tissue-selective carcinogens) should be considered to explain the high percentage of CpG mutations in some tumor types…”.

**Figure 3:**
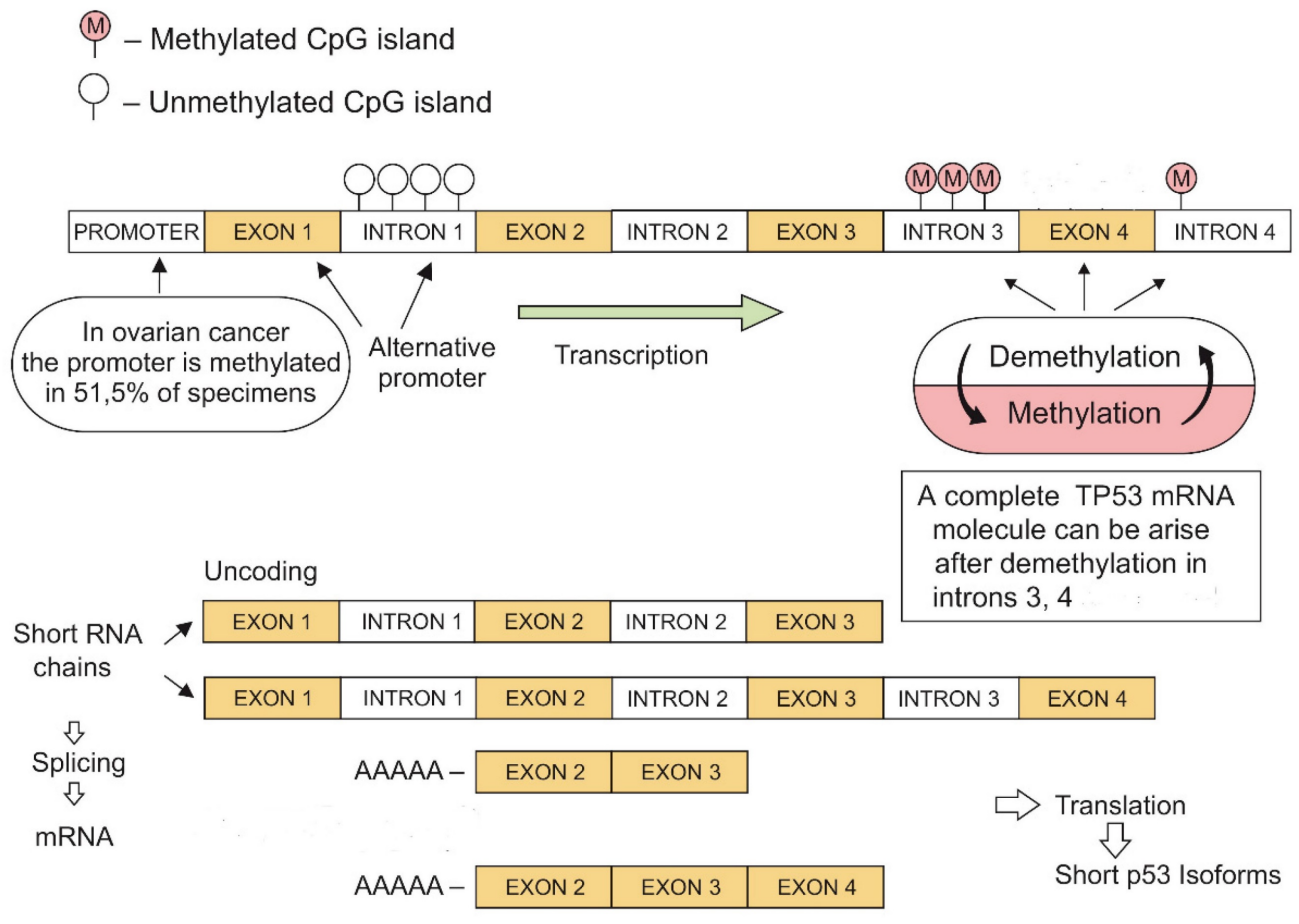
*TP53* demethylation-methylation mechanisms leading to the development of short mRNA isoforms (original image).

Our research showed methylation at the 3 and 4 introns of *TP53* in all analyzed cancers and healthy tissues. Moreover, introns 3 and 4 were also methylated in a human A2780 ovarian cancer cell line. The alternative promotor in *TP53* intron 1 was un-methylated and equally active in both ovarian tumors and healthy tissues. This observation may explain the positive expression of p53 in most tumors with a methylated promoter gene segment. Our findings may suggest the existence of an additional mechanism for regulating gene expression through the demethylation-methylation at *TP53* introns (Fig. 3).

The complete *TP53* mRNA molecule can arise only *via* demethylation of everyone's intragenic sections during gene transcription 11. The demethylation-methylation mechanism may result in the yield formation of short RNA strands that ensure conformational stability of the tetrameric p53 protein molecule and its domain activities (Fig. 2).

The expression of the human p53 may be controlled by two promoters, and differential regulation of these promoters plays an important role in the altered expression of the gene in both normal and transformed cells 20. It is well-known that methylation causes a change in the chromatin structure that prevents re-binding of regulatory proteins 31-32. It is generally accepted that high methylation of certain chromatin fragments is associated with its partial or even complete transcriptional inactivation. In mammals, about 5% of the cytosine residues are continuously methylated. In plants, however, the percentage of methylation is even higher, and it is usually around 30% of the CpG pairs. Interestingly, methylation does not occur in some organisms, for example, in *Drosophila melanogaster* 33. The level of expression of a gene is correlated with the amount of methylated DNA in the promoter sequence. Specific gene promoter hypermethylation has been reported in patients affected by lung, oral, bladder, colon, non-small-cell lung, head and neck squamous cell carcinoma, as well as in malignant mesothelioma 34-36.

Although these data are novel, our study has certain limitations. Firstly, we investigated *TP53* introns methylation only in advanced-stage OCs, and further research is necessary to analyze early-stage tumors as well. Secondly, it would be interesting to simultaneously compare introns and exons *TP53* methylation patterns to find out their roles, if any, apart from analyzing only the CpG pair methylation within the gene's promoter region. Finally, studies investigating the intron *TP53* methylation patterns in uncommon OC histotypes, for example, clear-cell or undifferentiated neoplasms, would also be of interest to explain the role of intron methylation in their carcinogenesis.

Our data on *TP53* intron methylation sheds new light on the mechanism of p53 activity for a better understanding of cancer biology. The study suggests the existence of an additional regulation rule of *TP53* activity that involves demethylation-methylation mechanisms. Intragenic methylation at introns 3 and 4 may overall help protecting *TP53* against damage by restrictases or viral DNA integration.

## Figures and Tables

**Table 1:** Clinical characteristic of the ovarian cancer patients.

	Ovarian cancer subtype	
	Endometrioid (n=40)	Serous (n=40)
	n (%)	n (%)
Patient age (years)		
<50	1 (2.5)	-
50-60	10 (25)	8 (20)
>60	29 (72.5)	32 (80)
Menopausal status		
pre-menopausal	2 (5)	1 (2.5)
post-menopausal	38 (95)	39 (97.5)
FIGO III		
A	2 (5)	3 (7.5)
B	20 (50)	16 (40)
C	18 (45)	21 (52.5)
Histological grade		
G1	-	-
G2	19 (47.5)	18 (45)
G3	21 (52.5)	22 (55)

**Table 2:** Primer sequences, product sizes, and CpG pairs investigated in introns 1, 3 and 4 of the *TP53* suppressor gene.

Intron	Primer sequence		Product size (bp)	CpG pairs	
	Forward	Reverse		Total	Investigated
1	5'-GGGTAAGTTTTTGATTGAATTTGAT-3'	5'-CAAAACTCCACTCCTCTACCTAAAC-3'	259	8	8
3	5'-ATTTTTTTTGGGGATGTAGAATTTT-3'	5'-ACACCACCATACCTAACTAATTTTT-3'	231	3	2
4*	5'-TTGTGTAGTTGTGGGTTGATTTTATAT-3'	5'-AAAAACCTAAAAACCCTAAACAACC-3'	193	11	1

## Abbreviations

cDNA: complementary deoxyribonucleic acid
FIGO: International Federation of Gynecology and Obstetrics
mRNA: messenger ribonucleic acid
OC: ovarian cancer
PCR: polymerase chain reaction
WHO: World Health Organization

## References

[1] American Cancer Society (2015). Cancer facts & figures. Atlanta, GA: American Cancer Society.

[2] Reid MB, Permuth JB, Sellers TA (2017). Epidemiology of ovarian cancer: A review. Cancer biology and medicine.

[3] Kurman RJ, Ellenson LH, Ronnett BM (2018). Editors. Blaustein's Pathology of the Female Genital Tract. Seventh Ed, Springer.

[4] Bourdon J-C (2007). p53 and its isoforms in cancer. British journal of cancer.

[5] Toledo F, Geoffrey M Wahl (2006). Regulating the p53 pathway: in vitro hypotheses, in vivo veritas. Nature reviews cancer.

[6] Vousden KH, David PL (2007). p53 in health and disease. Nature reviews molecular cellular biology.

[7] Semczuk A, Schneider-Stock R, Szewczuk W (2010). Prevalence of allelic loss at TP53 in endometrial carcinomas. Oncology.

[8] Code AJ, Dwight T, Gill AJ, Dickson K-A, Zhu Y, Clarkson A (2016). Assessing mutant p53 in primary high-grade serous ovarian cancer using immunohistochemistry and massive parallel sequencing. Scientific reports.

[9] Zhang Y, Cao L, Nguyen D, Lu H (2016). TP53 mutations in epithelial ovarian cancer. Translational cancer research.

[10] Semczuk A, Gogacz M, Semczuk-Sikora A, Jozwik M, Rechberger T (2017). The putative role of TP53 alterations and p53 overexpression in borderline ovarian tumors - correlation with clinicopathological features and prognosis: A mini-review. Journal of cancer.

[11] Carninci P, Sandelin A, Lenhard B (2006). Genome-wide analysis of mammalian promoter architecture and evolution. Nature genetics.

[12] Mattick JS, Gagen MJ (2001). The evolution of controlled multitasked gene networks: the role of introns and other noncoding RNAs in the development of complex organisms. Molecular biology evolution.

[13] Rose AB (2008). Intron-mediated regulation of gene expression. Current topics in microbiology and immunology.

[14] Louro R, Smirnova AS, Verjovski-Almeida S (2009). Long intronic noncoding RNA transcription: expression noise or expression choice?. Genomics.

[15] Bourdon J-C, Fernandes K, Murray-Zmijewski F (2005). p53 isoforms can regulate p53 transcriptional activity. Genes development.

[16] Reisman D, Greenberg M, Rotter V (1988). Human p53 oncogene contains one promoter upstream of exon 1 and second stronger promoter within intron 1. Proceeding of the national academy of science USA.

[17] Reisman D, Balint E, Loging WT, Rotter v, Almon E (1996). A novel transcript encoded within the 10-kb first intron of the human p53 tumor suppressor gene (D17S2179E) in induced during differentiation of myeloid leukemia cells. Genomics.

[18] Ribi S, Baumhoer D, Lee K, Edison, Teo ASM, Madan B (2015). TP53 intron 1 hotspot reaarrangments are specific to sporadic osteosarcoma and can cause Li-Fraumeni syndrome. Oncotarget.

[19] Marcel V, Tran PLT, Sagne C, Martel-Planche G, Vaslin L, Teulade-Fichou MP (2011). G-quadruplex structures in TP53 intron 3: role in alternative splicing and in production of p53 mRNA isoforms. Carcinogenesis.

[20] Luczak MW, Jagodzinski PP (2006). The role of DNA methylation in cancer development. Folia histochemica and cytobiologica.

[21] Sharma S, Kelly TK, Jones PA (2010). Epigenetics in cancer. Carcinogenesis.

[22] Koukoura O, Spandidos DA, Daponte A, Sifakis S (2014). DNA methylation profiles in ovarian cancer: Implication in diagnosis and therapy (Review). Molecular medicine reports.

[23] Tirado-Magallanes R, Rebbani K, Lim R, Pradhan S, Benoukraf T (2017). Whole genome DNA methylation: beyond genes silencing. Oncotarget.

[24] Matei D, Nephew KP (2020). Epigenetic attire in ovarian cancer: The Emperor's new clothes. Cancer research.

[25] Female Genital Tumours (2020). WHO Classification on Tumours. 5th Ed, Volume 4.

[26] Berek JS, Renz M, Kehoe S, Kumar L, Friedlander M (2021). Cancer of the ovary, fallopian tube, and peritoneum: 2021 update. International journal of gynaecology and obstetrics.

[27] Chmelarova M, Krepinska E, Spacek J, Laco J, Beranek M, Palicka V (2013). Methylation in the p53 promoter in epithelial ovarian cancer. Clinical and translational oncology.

[28] Chmelarova M, Kos S, Dvorakova E, Spacek J, Laco J, Ruszova E (2014). Importance of promoter methylation of GATA4 and TP53 genes in endometrioid carcinoma of endometrium. Clinical chemistry and laboratory medicine.

[29] Jha AK, Nikbakht M, Jain V, Sehgal A, Capalash N, Kour J (2012). Promoter hypermethylation of p73 and p53 genes in cervical cancer patients among north Indian population. Molecular biology reports.

[30] Tornaletti S, Pfeifer GP (1995). Complete and tissue-independent methylation of CpG sites in the p53 gene: implications for mutations in human cancers. Oncogene.

[31] Scoumanne A, Chen X (2008). Protein methylation: a new regulation of the p53 tumor suppressor. Histology histopathology.

[32] Ng S, Yue W, Oppermann U, Klose RJ (2009). Dynamic protein methylation in chromatin biology. Cellular and molecular life sciences.

[33] Lyko F, Ramsahoye BH, Jaenisch R (2000). DNA methylation in Drosophila melanogaster. Nature.

[34] Locke WJ, Guanzon D, Ma C, Liew YJ, Duesing KR, Fung KYC et (2019). al. DNA methylation cancer biomarkers: Translation to the clinic. Frontiers in genetics.

[35] Chmelarova M, Palicka V (2019). Epigenetics in cancer: a promising path to follow?. Clinical chemistry and laboratory medicine.

[36] Bhootra S, Jill N, Shanmugam G, Rakshit S, Sarkar K (2023). DNA methylation and cancer: transcriptional regulation, prognostic, and therapeutic perspective. Medical oncology.

